# The properties of absorbable scaffold harvested with human amniotic fluid stem cells on rat model: an innovation for pelvic reconstruction surgery

**DOI:** 10.1038/s41598-024-63375-3

**Published:** 2024-06-03

**Authors:** Tsia-Shu Lo, Yi-Pin Chen, Fazlin Harun, Steven W. Shaw, Yi-Hao Lin

**Affiliations:** 1 Division of Urogynecology, Department of Obstetrics and Gynecology, Linkou, Chang Gung Memorial Hospital, Linkou Medical Center, 5, Fu-Hsin Street, Kwei-shan, 333 Taoyuan, Taiwan; 2 Department of Obstetrics and Gynecology, Chang Gung Memorial Hospital, Keelung Medical Center, Keelung, Taiwan; 3 Department of Obstetrics and Gynecology, Medical Center, Chang Gung Memorial Hospital, Taipei, Taiwan; 4 School of Medicine, Chang Gung University, Taoyuan, Taiwan; 5 Department of Obstetrics and Gynecology, Women and Children Hospital (Hospital Tunku Azizah), Kuala Lumpur, Malaysia

**Keywords:** Stem cells, Medical research, Urology

## Abstract

The current practice of restoring the anatomical structure in the treatment of pelvic floor dysfunction includes implantation of synthetic sling, which carries potential complications. This study aimed to develop biological substitutes to improve tissue function using scaffolds as a support to the host cells, through formation of new tissue. Human amniotic fluid stem cells (hAFSCs) were seeded on synthetic mesh-scaffold of AlloDerm Regenerative Tissue Matrix (RTM), Poly-DL-lactico-glycolic acid (PLGA) mesh (VICRYL) and Polydioxanone (PDS) meshes. In vitro study evaluates the metabolic activity of hAFSCs seeded mesh-scaffolds. In vivo study involving Sprague–Dawley rats was performed by assigning into 7 groups of sham control with fascia operation, AlloDerm implant, PDS implant, PLGA implant, AlloDerm harvest with hAFSC (AlloDerm-SC), PDS harvest with hAFSC(PDS-SC) and PLGS harvest with hAFSC (PGLA-SC). In vitro study reveals cell viability and proliferation of hAFSC on mesh scaffolds varies between meshes, with AlloDerm growing the fastest. The biomechanical properties of tissue-mesh-complex tension strength declined over time, showing highest tension strength on week-1, deteriorated similar to control group on week-12. All hAFSC-seeded mesh provides higher tension strength, compared to without. This study shed the potential of synthetic mesh as a scaffold for hAFSC for the surgical treatment of pelvic floor dysfunction.

## Introduction

Pelvic floor dysfunction, especially pelvic organ prolapse (POP) is an aging-related disease, as the result of weakening pelvic floor support. Reinforcement of this debilitated pelvic floor support usually requires surgical repair by natural or synthetic material, such as autologous fascia or surgical meshes^1^.

The current practice of restoring the anatomical structure in the treatment of pelvic floor dysfunction involve the implantation of synthetic sling materials, predominantly polypropylene (PPL) meshes. However, these procedures carries significant long-term complications, including erosion, pain, and voiding and sexual dysfunction^2^. These issues have led to litigation problems and regulatory actions by the US Food and Drug Administration (FDA), including withdrawals and restrictions on mesh usage.

Various synthetic materials, including polyester, polytetrafluoroethylene (PTFE) and polyvinylidene fluoride (PVFD) have been utilized in surgical meshes^3^. However, each material presents its own set of challenges, contributing to high rates of mesh removal following surgery. Efforts to improve mesh materials includes bioactive coating and the use of different polymers such as poly(lactic-co-glycolic) acid (PGLA) and poly-L-lactic acid (PLA). Despite promising result in vivo studies, these materials have shown mechanical and biological failures when used in pelvic floor repair^4^.

There is a pressing need for better synthetic materials to support the pelvic floor, but progress is hindered by a lack of basic research on biomechanical properties and safety studies, specifically on the weakened pelvic tissues^5^. Predicting or modelling the mechanical pressure of the pelvic floor to ensure prosthesis stability is also challenging. Additionally, materials need to be able to interact with cells and promote fibroblast ingrowth and extracellular matrix production to prevent failure^5^.

Advancement of tissue engineering offer promise for developing biological substitutes to restore tissue function. Scaffolds can be developed to support the host cells, promoting their differentiation and proliferation to form new tissue^6^, allowing for the creation of functional tissue.

This study is based on 4 concepts.

1. Absorbable Meshes: Synthetic absorbable meshescomposed of polyglactin or polyglycolic acid (PGA) were once used for Pelvic Reconstructive Surgery (PRS). However, their poor long-term tensile strength and high recurrence^7,8^ rates limit their reconstructive efficiency.
2. Absorbable meshes and Human Amniotic Stem (hAFSC): hAFSC are ideal seed cells for tissue regeneration due to their wide availability, biological characteristics, and low risk of immune rejection. The combination of synthetic absorbable mesh with hAFSCs has shown promise in tissue regeneration.
3. Scaffold environment for human Amniotic Fluid Stem Cell (hAFSC) : Modifications to transvaginal meshes, such as combining non-absorbable material, aim to improve biocompatibility and mechanical stability^9,10^. Coating meshes with hAFSC can achieve the balance of mechanical stability and biocompability.
4. Scaffold degeneration: The chosen biomaterial should provide temporary mechanical support while allowing tissue growth and degradation over time^11^.

In this study, three absorbable materials, Acellular dermal matrix (ADM) AlloDerm Regenerative Tissue Matrix (RTM) (SELECT™, BioHorizons, Birmingham, AL); Biodegradable poly-DL-lactico-glycolic acid (PGLA) mesh (VICRYL^®^, polyglactin 910) woven Mesh, Ethicon, Bridgewater, NJ) and Polydioxanone (PDS) mesh (DuraSorb, SIA, Chicago, Ill) were compared. The hypothesis is that utilizing commercially available absorbable mesh as scaffold with hAFSC cultivation, may provide both tissue compatibility, and appropriate tension to support pelvic floor.

## Materials and methods

### Ethical approval

Experimental protocols and procedures were approved by Chang Gung Memorial Hospital’s Institutional Animal Care and Use Committee (No. 2019062002), Institutional Board Review (IRB: 201800954B0) and funded by the National Science and Technology Council Grants (MOST 107-2314-B-182A-103-). The study period took place from August 1^st^, 2018 to January 31st, 2020.

All procedures involving humans were carried out in accordance with relevant guidelines and regulations, and approved by Institutional Board Review Chang Gung Memorial Hospital. Informed consent was obtained from all participants/donor.

All experimental procedures were performed under the supervision of a licensed veterinarian, in a manner consistent with the regulations of the National Institute of Health of Taiwan. All animal related procedures were approved by the Institutional Animal Care and Use Committee of Chang Gung Memorial Hospital (IACUC Approval No.: CGMH2019062002). All methods involving animals are reported in accordance with ARRIVE guidelines.

### Isolation and characterization of hAFSC for transplantation

The hAFSCs were obtained from freshly collected amniotic fluid by routine amniocentesis from healthy pregnant donors at 15–20 gestational weeks. Cells were cultured in StemPro® MSC Serum free medium supplemented with 10% fetal bovine serum (Invitrogen, Carlsbad,CA) and incubated at 37 °C with 5% carbon dioxide. Culture medium was changed every 3–4 days. The specific surface antigens of hAFSCs were characterized using flow cytometry analyses. The cultured cells were trypsinized and stained with phycoerythrin (PE)-conjugated antibodies against CD90 (BD PharMingen,CA). The cells were analyzed using the Calibur flow cytometer (Becton Dickinson, Heidelberg, Germany). Passage 4 to 6 hAFSCs were collected and prepared to a final concentration of 3 × 10^6^ cells/0.3 mL Phosphate Buffer Solution (PBS). Thereafter, 3 × 10^6^ hAFSCs were seeded on a sterile mesh-scaffold and cultured for 3 days prior transplantation. This is accordance to the previous work by Liang et al.^12^.

### In vitro and in vivo study: mesh and hAFSC

**Figure 1:**
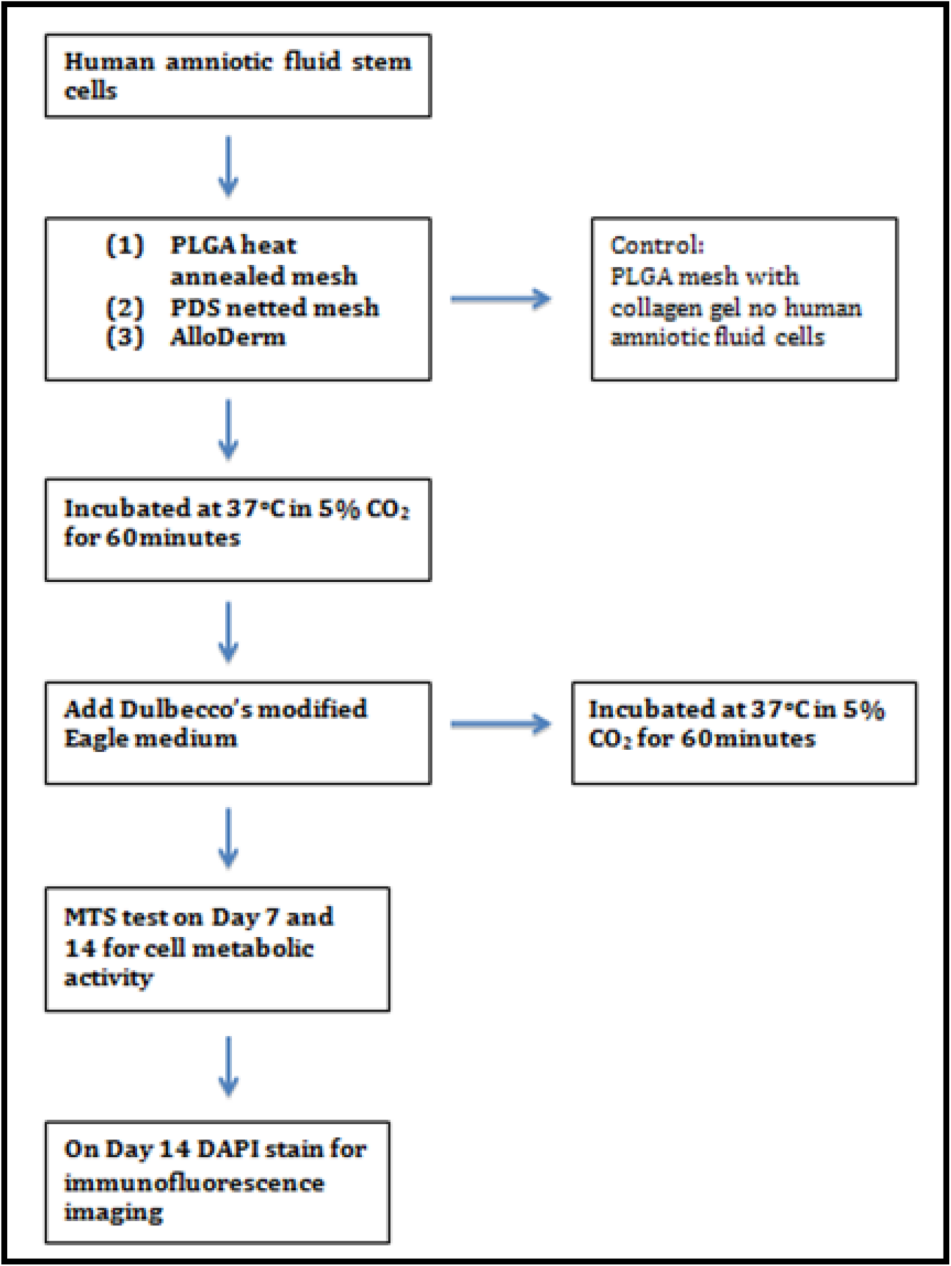
Flow Chart In Vitro.

Three types of absorbable materials were compared, comprising AlloDerm RTM; PLGA mesh (VICRYL^®^) and PDS mesh. The characteristic of each mesh is displayed in Table 1. Cell line from amniotic fluid stem cells was cultivated with basic fibroblast growth (bFGF). To measure the cell’s ability to proliferate, EdU Assay (Click-iT®EdU Assay, Invitrogen, Life Technologies Corporation, Carlsbad, CA, USA) is incubated with hAFSC, and meshes-harvested with hAFSc. EdU Assay (5-ethynyl-2’-deoxyuridine) works as a nucleoside analog of thymidine and is incorporated into DNA during active DNA synthesis. Procedure was performed in accordance to protocol (Supplementary, S1). DNA staining is performed for imaging and analysis. hAFSCs seeded mesh-scaffold were incubated at 37 °C in 5% CO2 for 60 min, followed by Dulbecco’s modified Eagle medium. MTS (5-(3-carboxymethoxyphenyl)-2-(4,5-dimethyl-thiazoly)-3-(4-sulfophenyl) tetrazolium, inner salt assay)^13^ colorimetric assay test were conducted on day 7 and day 14 for cell metabolic activity (Fig. 1). DAPI (Santa Cruz Biotechnology, Santa Cruz, CA, USA) Stain for immunofluorescence imaging were also conducted on day 14. The most suitable mesh with hAFSC growth was determined by immunofluorescence assay and scanning electron microscopy (SEM) via LIVE/DEAD^®^ Viability/cytotoxicity Kit on day 7 and day 14. SEM enables direct microscopic imaging of the material properties on the surface sample, that offers adjustable magnification and large field depth. In this study, spot charge-coupled device color digital camera (Olympus DP72, Tokyo, Japan) was used to obtain immunohistochemistry images under 20 × objective (Olympus BX-51, Tokyo, Japan) and immunofluorescence under Leica TCS SP8X confocal laser scanning microscope (Leica Microsystem, Heidelberg, Germany) with appropriate filters for DAPI. Camera was interfaced with Image-Pro Plus Software (Media Cybernetics, Silver Spring, MD, USA). This is in accordance as previous study conducted by Liang et al.^14^.

**Table 1:** Characteristic of Mesh.

Characteristics	AlloDerm	PDS Mesh	PLGA mesh
Material	Acellular dermal matrix	Polydioxanone	Polyglactin
Type of thread	–	Monofilament	Multifilament
Thread thickness	–	3–0	3–0
Type of mesh	–	Woven	Woven
Size of elements, mm	0.9–1.6	2.0–2.5	2.0–2.5
Tensile strength, MPa	1.5 ± 0.33	5.01 ± 0.72	8.76 ± 0.97
Porosity, %	0	85.8	82.8
Expected resorption time, days	70	170–250	60–90

### In vivo study: animal preparation

Total of 28 Sprague–Dawley rats, with the mean age 12.3 ± 1.7 weeks old and weighing 298.2 ± 27.1 g were treated and cared for under the supervision of a licensed veterinarian, in a manner consistent with the regulations of the National Institute of Health of Taiwan. All animal related procedures were approved by the Institutional Animal Care and Use Committee of Chang Gung Memorial Hospital (IACUC Approval No.: CGMH2019062002).

Seven groups were assigned: [1] sham control group with fascia operation; [2] AlloDerm implant; [3] PDS implant; [4] PLGA implant; [5] AlloDerm harvest with hAFSC (AlloDerm-SC); [6] PDS harvest with hAFSC (PDS-SC); and [7] PGLA harvest with hAFSC (PGLA-SC).

### Surgical procedure

**Figure 2:**
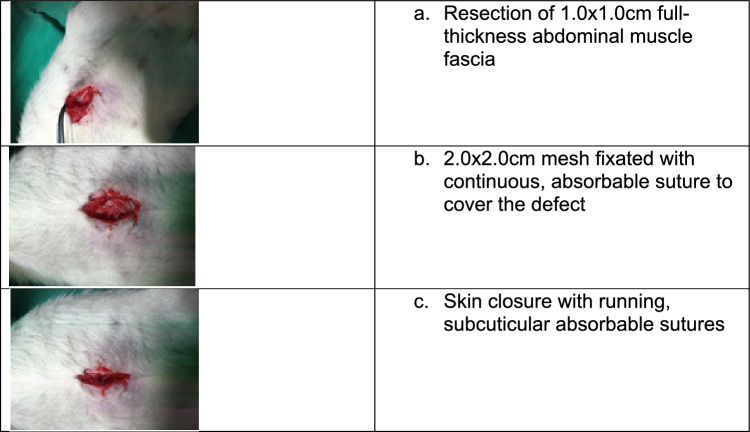
Surgical Procedure.

Rats were anesthetized with 2% Isoflurane^®^ mask inhalation. An abdominal midline incision of 4 cm and subcutaneous blunt dissection to muscle were made. A 1.0 × 1.0 cm full-thickness abdominal muscle fascia resected. Mesh measuring 2.0 × 2.0 cm was fixated with continuous, absorbable suture (Polygactin, Vicryl 3/0) to cover the defect. Skin was then closed with running subcuticular absorbable sutures (Vicryl 3/0) (Fig. 2). Sham control group with fascia operation underwent abdominal muscle fascia resection without mesh implantation. In the present study, the number of control rats was reduced in compliance with IACUC’s recommendation. Total of 12 rats (week 1), 4 rats (week 2) and 12 rats (week 12) were sacrificed at respective weeks. The tissues were harvested at 12 weeks, and meshes were retrieved for tensile properties characteristic and immuno-histological examination.

### Assessment of mechanical properties of the scaffold

**Figure 3:**
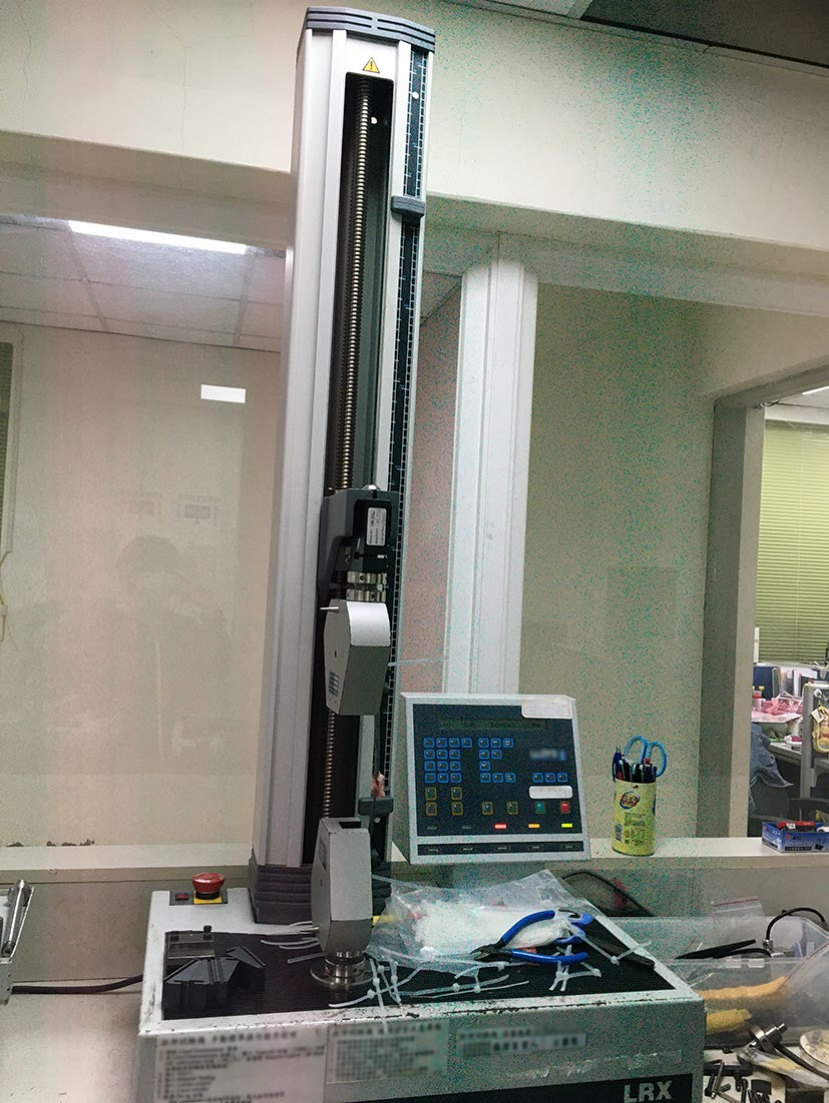
Tensiometry for mechanical properties of the scaffolds.

The mechanical properties of three absorbable mesh with and without AFSC meshes were estimated utilizing tensile test equipment (Lloyd, Ametek, Berwyn, PA, USA) (Fig. 3). The maximum strengths of three absorbable mesh with and without AFSC were compared. The stretching speed was set at 100 mm/min and the ultimate load and deformation were recorded.

### Euthanasia/Sacrifice methods

The rats were euthanized with 3% isoflurane and then decapitated, in accordance and manner consistent with the regulations of the National Institute of Health of Taiwan and Institutional Animal Care and Use Committee of Chang Gung Memorial Hospital.

### Statistical analysis

Sample size calculation was done by using crude method based on law of diminishing return with the equation of E = total number of animals-total number of groups. After the calculation with (7 groups × 6 rats/group) – (7groups) = 35, suggesting the sample size for this study was adequate, and 6 rats were used for each group^15^. The data were analyzed and expressed as mean ± SD for continuous variables. Continuous data were compared among the groups by using one-way analysis of variance. To evaluate the effect of hAFSC among groups, chi-square test was performed with Fisher’s exact test. Probability value of < 0.05 are statistically significant.

### Ethics statement and trial registration

All procedures involving humans were carried out in accordance with relevant guidelines and regulations, and approved by Institutional Board Review Chang Gung Memorial Hospital. Informed consent was obtained from all participants/donor. All experimental procedures were performed under the supervision of a licensed veterinarian, in a manner consistent with the regulations of the National Institute of Health of Taiwan. All animal related procedures were approved by the Institutional Animal Care and Use Committee of Chang Gung Memorial Hospital (IACUC Approval No.: CGMH2019062002). All methods involving animals are reported in accordance with ARRIVE guidelines.

## Results

### In vitro and in vivo study: mesh and hAFSC

**Figure 4:**
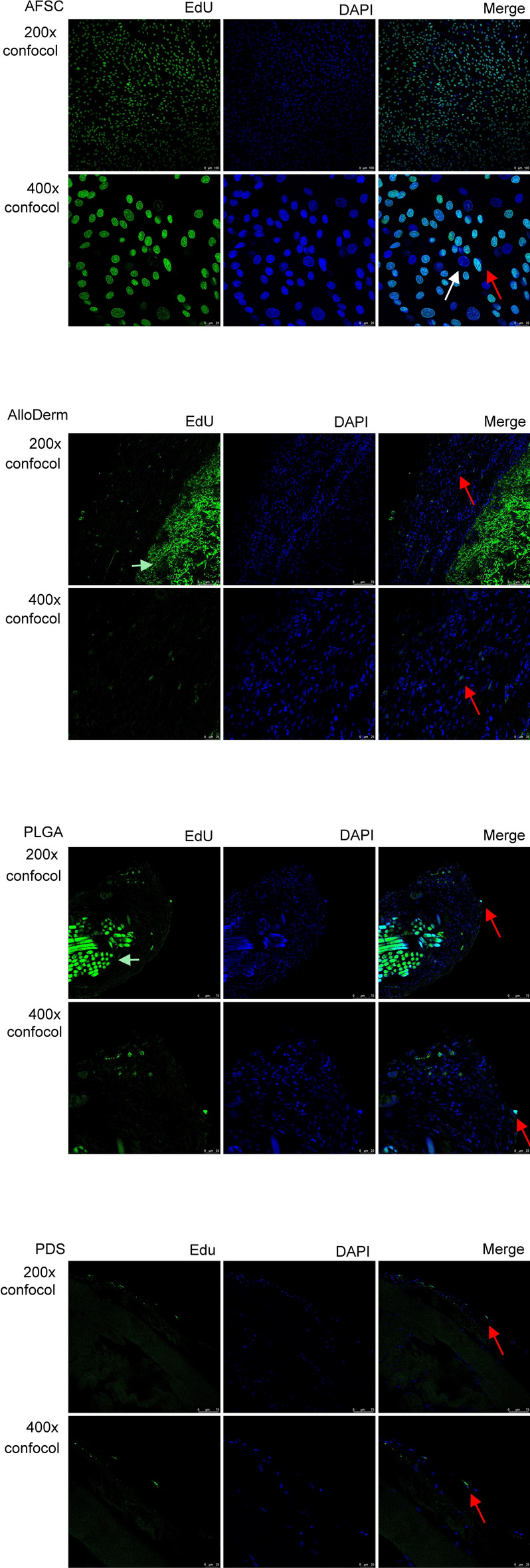
EdU Cell variability assay’s in vivo. *AlloDerm* AlloDerm Regenerative Tissue Matrix, *PDS* Polydioxanone mesh, *PGLA* poly-DL-lactico-glycolic acid, *EdU* 5-ethynyl-2´-deoxyuridine (Click-iT™ EdU Cell Proliferation Kit; Life Technologies Corporation, Ca, USA); AFSC, amniotic fluid stem cell; White arrow, without EdU uptake cell. Red arrow, with EdU uptake cell. Blue arrow, mesh fiber.

EdU uptakes confirms the presence of cell’s proliferation which is fundamental in quantifying and assessing cell variability. All three types of meshes shows positive finding of cell proliferation in vivo (Fig. 4).

**Figure 5:**
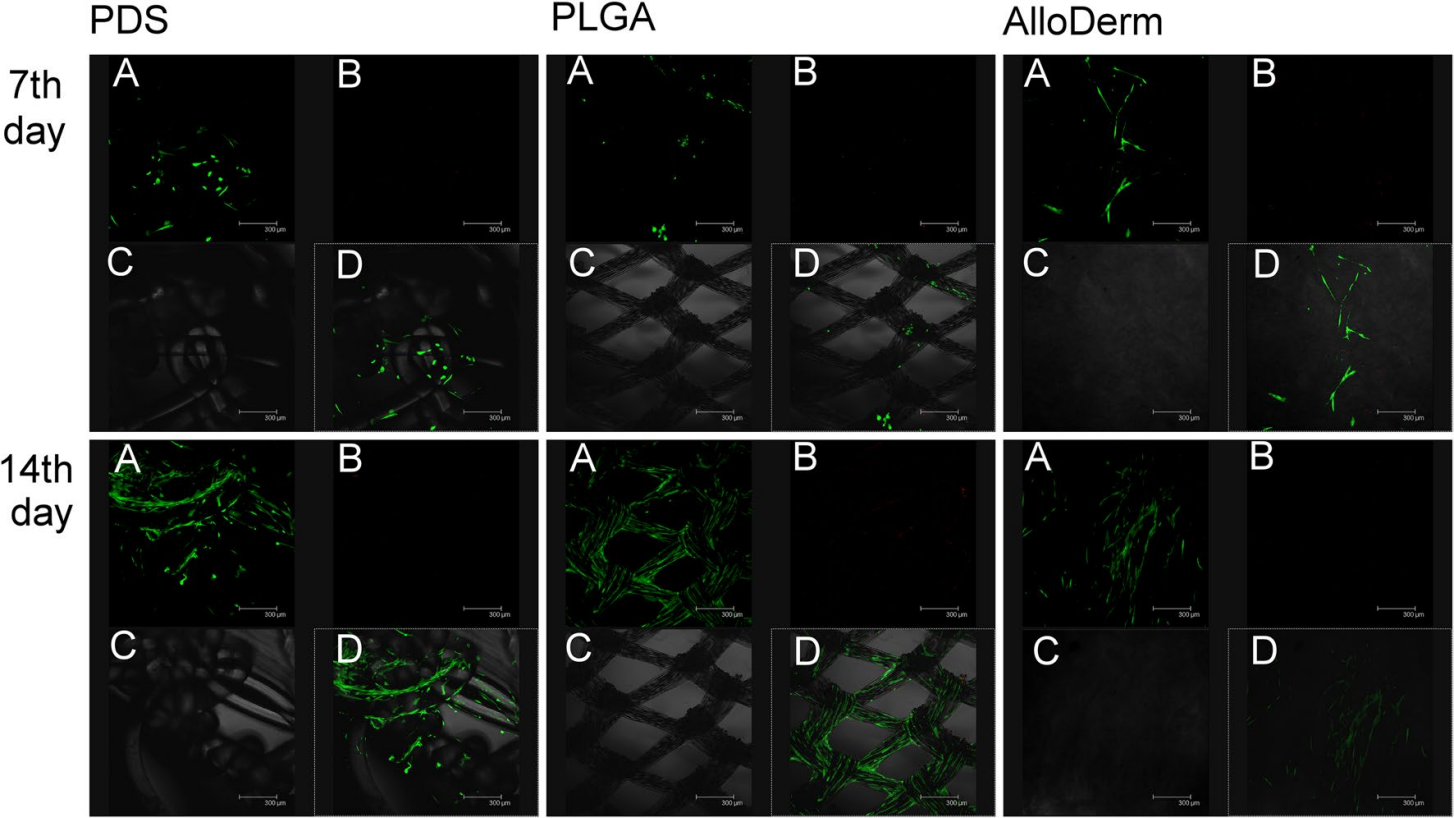
DAPI Stain for immunofluorescence imaging in vitro for hAFSC. *hAFSCs* human amniotic fluid stem cells, *PDS* Polydioxanone mesh, *PGLA* Biodegradable poly-DL-lactico-glycolic acid, *AlloDerm* acellular dermal matrix; *DAPI* 4′,6-diamidino-2-phenylindole (Thermo, USA); The green fluorescence represents living cells of the amniotic fluid stem cell line. (**A**) The red fluorescence represents death cells of the amniotic fluid stem cell line. (**B**) LIVE/DEAD^®^ Cell Viability Assays (Thermo, USA). *SEM* Scanning Electron Microscope (**C**).

The hAFSC cell attachment growth and viability were also observed using the LIVE/DEAD^®^ Viability/ Cytotoxicity Kit at day 7 and day 14 on all 3 meshes, which served as the cytoskeleton. Green fluorescence represents mitochondrial activity of living cells of the amniotic fluid stem cell line, among which AlloDerm grows fastest and most easily (Fig. 5). The cell number increased continuously in each mesh upon observation on day 14. However, the cell proliferation rate differs among meshes at each point of time. The result indicated that the cell viability and proliferation of hAFSC on mesh scaffolds are different between meshes. It is also observed that hAFSC cell growth in PDS and PGLA meshes only grow on the surface of cytoskeleton, compared to spread growth found on AlloDerm mesh.

### In vivo study

**Figure 6:**
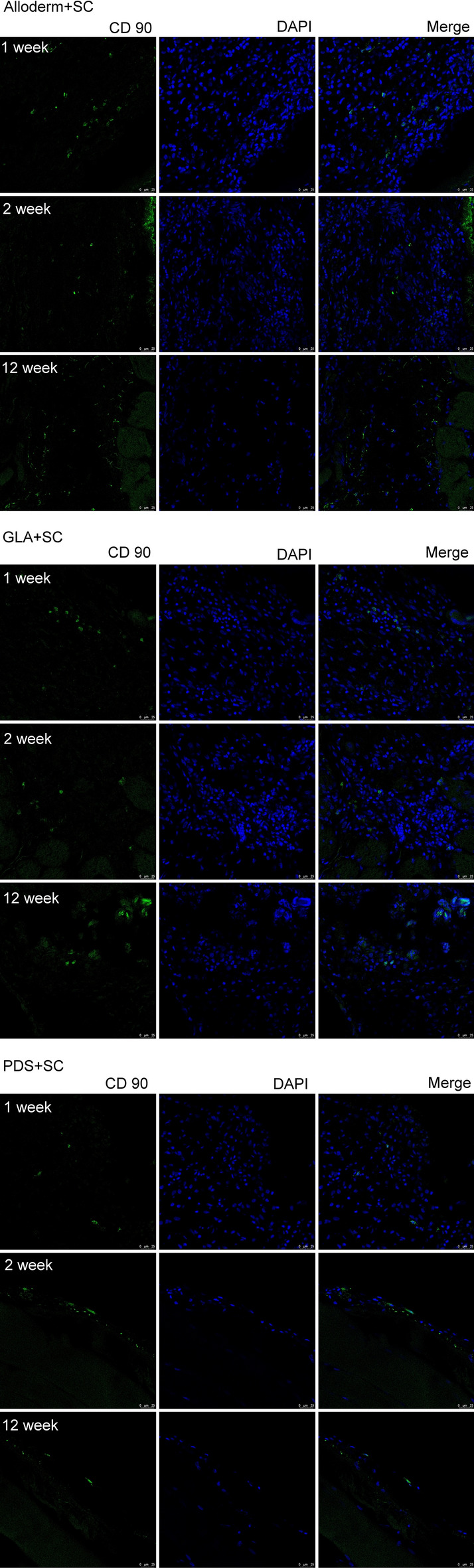
DAPI Stain for immunofluorescence Imaging in vivo. *AlloDerm* AlloDerm Regenerative Tissue Matrix, *AlloDerm* + *SC* AlloDerm harvest with hAFSC, *PDS* Polydioxanone mesh, *PDS* + *SC* PDS harvest with hAFSC, *PGLA* poly-DL-lactico-glycolic acid, *PGLA* + *SC* PGLA harvest with hAFSC, *hAFSCs* human amniotic fluid stem cells, *DAPI* 4′,6-diamidino-2-phenylindole; *The green fluorescence represents living cells of the amniotic fluid stem cell line.

All 28 rats survived the surgery with no post-operative complications such as wound dehiscence or mesh exposure. Stem cell evaluation was conducted on week 0 (6 rats), week 1(12 rats), week 2 (4 rats) and week 12 (12 rats). The engraftment effect of hAFSC on the meshes was evaluated by immunofluorescence study. Surface markers of stem cells in CD90-positive hAFSCs were present in all meshes in week-1, with the highest seen in Alloderm, eventually lower by week-12 (Fig. 6).

### Histopathology

Histopathological analysis of mesh showed inflammation, fibrosis, and foreign body reaction for all group. The mesh group rats showed larger areas of inflammation as compared to the sham group rats.

### Biomechanical properties

Table 2 shows the tension strength of tissue-mesh-complex variation with time. In week 1, all hAFSC-seeded meshes show higher tension strength in comparison to its own mesh groups. Highest tension strength is seen in PDS-SC (2.61 ± 0.05), with the fascia control reference of (0.49 ± 0.44). While in week 2, all groups show better outcome in strength in comparison to the control group of sham (fascial), except in PGLA and PGLA-SC groups, in which the tension strength lowered similar to the fascial tension strength.

**Table 2:** Biomechanical Properties: Tension strength of tissue-mesh complex variation with time.

	1-week n = 6	p value**	2-week n = 2	12-week n = 6	p value***
Fascial	0.49 ± 0.44*	Reference	x	x	
AlloDerm-SC	2.05 ± 0.09	< 0.001	1.84 ± 0.36	0.33 ± 0.15	0.404
AlloDerm	1.91 ± 0.42	< 0.001	1.17 ± 0.06	0.25 ± 0.08	0.214
PDS-SC	2.61 ± 0.26	< 0.001	1.42 ± 0.11	0.59 ± 0.14	0.642
PDS	2.22 ± 0.05	< 0.001	1.17 ± 0.02	0.51 ± 0.06	0.940
Vicryl-SC	0.86 ± 0.07	0.075	0.49 ± 0.47	0.39 ± 0.17	0.539
Vicryl	0.54 ± 0.12	0.214	0.18 ± 0.03	0.29 ± 0.19	0.313

*AlloDerm* AlloDerm Regenerative Tissue Matrix, *AlloDerm-SC* AlloDerm harvest with hAFSC, *PDS* Polydioxanone mesh, *PDS-SC*, PDS harvest with hAFSC, *PGLA* poly-DL-lactico-glycolic acid, *PGLA-SC* PGLA harvest with hAFSC, *hAFSCs* human amniotic fluid stem cells.

Data listed as mean ± standard deviation (95% confidence interval).

**Comparison between Control and 1 week.

***Comparison between Control and 12 week.

Independent-samples t test.

**Figure 7:**
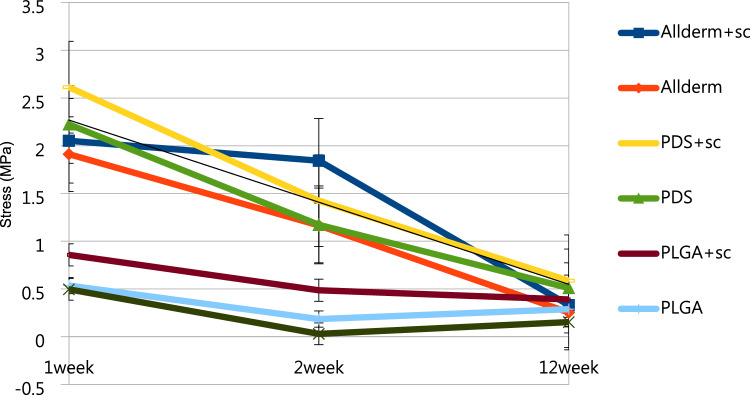
Tensile properties of PDS, polydioxanone mesh; *PLGA* poly-DL-lactico-glycolic acid(PGLA) mesh, *AlloDerm* acellular dermal matrix. *AlloDerm* AlloDerm Regenerative Tissue Matrix, *AlloDerm* + *SC* AlloDerm harvest with hAFSC, *PDS* Polydioxanone mesh, *PDS* + *SC* PDS harvest with hAFSC, *PGLA* poly-DL-lactico-glycolic acid, *PGLA* + *SC* PGLA harvest with hAFSC, *hAFSCs* human amniotic fluid stem cells.

The strength decreases over time from week 1 to week 12 in all group, in which the strength at week 12, appears similar or lower to the controlled fascial group. Despite that, it is noted at week 12, the hAFSC-seeded meshes has served better than without hAFSC in all mesh groups (Fig. 7).

## Discussion

The fundamental element for successful tissue engineering lies in the selection of biomaterial combined with the appropriate cells and growth-inducing factors^16^. The biological scaffold material for tissue engineering must fulfill several criteria^17^: (1) exhibiting good biocompatibility, to facilitate cell adhesion, and proliferation, without toxicity or immunogenicity; (2) being biodegradable; (3) possessing adequate mechanical strength to guide tissue generation; and (4) featuring a suitable porosity and pore size.

For pelvic floor repair meshes, which necessitate specific mechanical properties and biocompatibility, a composite mesh comprising synthetic and natural polymers appears to offer the most promising combination for ideal pelvic floor mesh material. This study applies hAFSC to enhance cell adhesions and promotes tissue remodeling, while synthetic polymers contribute mechanical strength.

Our study confirmed the feasibility of incorporating hAFSC on commercially available absorbable synthetic mesh kit. However, the adherence and growth of hAFSC on selected mesh varied according to textile, porosity, and tensile characteristic of the mesh (Table 1). In vivo study is demonstrating positive variability in cell presence, indicating biocompatibility across all three meshes.

Through in vitro investigation, hAFSC attachment and growth were most robust on AlloDerm, attributed to its acellular dermal matrix properties supporting revascularization and reduced inflammatory response, promoting regeneration. Meshes with large pore size exhibited poor adherence for hAFSC growth, highlighting the significant of scaffold porosity and pore size in facilitating cell infiltration and nutrient diffusion^18^.

Tension strength of tissue-mesh complex, was initially highest in PDS, followed by AlloDerm and lastly PLGA, with all hAFSC-seeded meshes outperforming their original counterparts. However, over time, tension strength decreases, correlating with mesh absorbability rates.

Although hAFSC accelerate wound healing, mesh degradation over time weakens the tissue-mesh complex. Achieving balance between mesh degradation and stem cell growth is crucial, as premature absorption compromises tissue support^19^, healing takes time. However, over time, despite tissue healing progresses, the absorbable mesh.

Despite tissue fibrogenesis and mesh degradation asynchrony, hAFSC-seeded meshes demonstrated superior performance compared to unseeded meshes in all groups, suggesting an additive effect on scaffold strength.

While tensile strength alone does not is predict success in PRS^20^, remodeling in the host is pivotal, especially for biocompatible absorbable materials^21^.

This preliminary study integrates hAFSC with absorbable meshes, offering insight into their biomechanical properties and highlighting the need for further clinical trials to optimize scaffolding systems for PRS. To our knowledge this is the first study that incorporate hAFSC with absorbable meshes as a scaffold for the prospect of pelvic floor reconstructive surgery for comparison. The biomechanical properties evaluated offers better understanding in tissue-mesh complex.

The present study has some limitations. This study utilizes a single concentration of hAFSC, it is possible with a higher concentration, a better outcome could be achieved. The biochemical properties were evaluated up to 12 weeks, chances of better outcome in a longer duration can take place owing to tissue fibrosis. This animal study uses full-thickness abdominal wall defect models, rather than sites associated with pelvic floor dysfunction, which limits their effectiveness for evaluating mesh implants. The transitional challenge from rats’ abdominal wall is different from the elastic and fibrous tissue of vaginal wall, making evaluation of regenerative properties under optimal biological condition harder. Another factor to take in to consideration is unsuitability of hAFSC in rat, in which consideration of using rat AFSC^22^ may alter the result, including toxicity study to evaluate the possibility of leachable from scaffolds. Nevertheless, the use of rat model was suitable to assess the regenerative properties of different mesh types and its response following mesh implantation. In this study, the tensile properties were compared between scaffolds, it would be interesting to compare the mechanical properties of scaffold with the available PPL meshes to predict the outcome in clinical setting.

The ideal scaffolding system for PRS should not only able to withstand external mechanical forces but also provide biocompatibility. Unfortunately, none of the tissue-mesh complex in this study can produce a scaffold that can efficiently meet all these criteria. It is nevertheless an initial step to developing a better scaffolding system, and further increase the chance of clinical translation. We proposed more clinical trials to be conducted in the attempt to find the ideal scaffolding system for PRS.

In conclusion, our findings indicate that commercially available synthetic mesh kits could serve as a role of scaffold for hAFSC, with AlloDerm scaffold being the most conducive for hAFSC cultivation. Despite the absence of consistent strength enhancement by week 12, this study underscores the potential of tissue engineering in advancing surgical treatment for pelvic organ prolapse.

### Supplementary Information

Supplementary Information.

## Data Availability

The data of this study can be provided by the corresponding author upon reasonable request.
