# Supplementary material for: The properties of absorbable scaffold harvested with human amniotic fluid stem cells on rat model: an innovation for pelvic reconstruction surgery
Source: Sci Rep. 2024 Jun 3;14:12750. doi: 10.1038/s41598-024-63375-3 (PMC11148079; doi:10.1038/s41598-024-63375-3)
Supplement: Supplementary file 1 — Supplementary Information. [file 41598_2024_63375_MOESM1_ESM.pdf]

Click-iT® EdU Imaging Kits

Search the handbook

Buy Now

Table 1. Contents and storage information.

Material	C10337	C10338	C10339	C10340	Concentration	Storage*
EdU (Component A)	5 mg	5 mg	5 mg	5 mg	NA	• 2–6°C<br>• Desiccate<br>• Protect from light<br>• DO NOT FREEZE
Alexa Fluor® azide (Component B)	1 vial (Alexa Fluor® 488)	1 vial (Alexa Fluor® 555)	1 vial (Alexa Fluor® 594)	1 vial (Alexa Fluor® 647)	NA	
Dimethylsulfoxide (DMSO, Component C)	4 mL	4 mL	4 mL	4 mL	NA	
Click-iT® EdU reaction buffer (Component D)	4 mL	4 mL	4 mL	4 mL	10X solution containing Tris-buffered saline	
$CuSO_4$ (Component E)	1 vial	1 vial	1 vial	1 vial	100 mM aqueous solution	
Click-iT® EdU buffer additive (Component F)	400 mg	400 mg	400 mg	400 mg	NA	
Hoechst 33342 (Component G)	35 µL	35 µL	35 µL	35 µL	10 mg/mL in water	

*These storage conditions are appropriate when storing the entire kit upon receipt. For optimal storage conditions for each component, see labels on the vials. When stored as directed, this kit is stable for 1 year.
NA = Not applicable.

Number of assays: Sufficient material is supplied for 50 coverslips based on the protocol below.

Approximate fluorescence excitation/emission maxima, in nm: Alexa Fluor® 488 azide**:** 495/519; Alexa Fluor® 555 azide**:** 555/565; Alexa Fluor® 594 azide: 590/615; Alexa Fluor® 647 azide: 650/670; Hoechst 33342: 350/461, bound to DNA.

Introduction

Measuring a cell's ability to proliferate is a fundamental method for assessing cell health, determining genotoxicity, and evaluating anti-cancer drugs. The most accurate method of doing this is by directly measuring DNA synthesis. Initially this was performed by incorporation of radioactive nucleosides[3] (for example, H-thymidine). This method was replaced by antibody-based detection of the nucleoside analog bromo-deoxyuridine (BrdU). The Click-iT® EdU Assay from Invitrogen is a novel alternative to the BrdU assay. EdU (5-ethynyl-2´-deoxyuridine) provided in the kit is a nucleoside analog of thymidine and is incorporated into DNA during active DNA synthesis.[1] Detection is based on a click reaction,[2-5] a copper-catalyzed covalent reaction between an azide and an alkyne. In this

application, the EdU contains the alkyne and the Alexa Fluor® dye contains the azide. The advantages of the Click-iT® EdU labeling are readily evident while performing the assay. The small size of the dye azide allows for efficient detection of the incorporated EdU using mild conditions. Standard aldehyde-based fixation and detergent permeabilization are sufficient for the Click-iT® detection reagent to gain access to the DNA. This is in contrast to BrdU assays that require DNA denaturation (typically using HCl or heat or digestion with DNase) to expose the BrdU so that it may be detected with an anti-BrdU antibody (Figure 1).

The denaturation step in the BrdU protocol can disrupt dsDNA integrity, which can affect nuclear counterstaining, and can also destroy cell morphology and antigen recognition sites. In contrast, the EdU assay kit is not only easy to use, but is fully compatible with DNA staining, including dyes for cell cycle analysis. The EdU assay kit can also be multiplexed with surface and intracellular marker detection using antibodies (see Table 2 for details). Finally, unlike the BrdU assay, which relies upon antibodies which can exhibit nonspecific binding, the Click-iT® EdU assay uses bioorthogonal (biologically unique) moieties, producing low backgrounds and high detection sensitivities.

The kit contains all of the components needed to label and detect the incorporated EdU as well as perform cell cycle analysis on samples from adherent cells (Figure 2). For cell cycle analysis, the kit is supplied with blue fluorescent Hoechst 33342 dye. The kit includes sufficient reagents for labeling 50, 18 × 18 coverslips using 500 µL of reaction buffer per test. For the latest information on Click-iT® EdU, visit www.lifetechnologies.com.

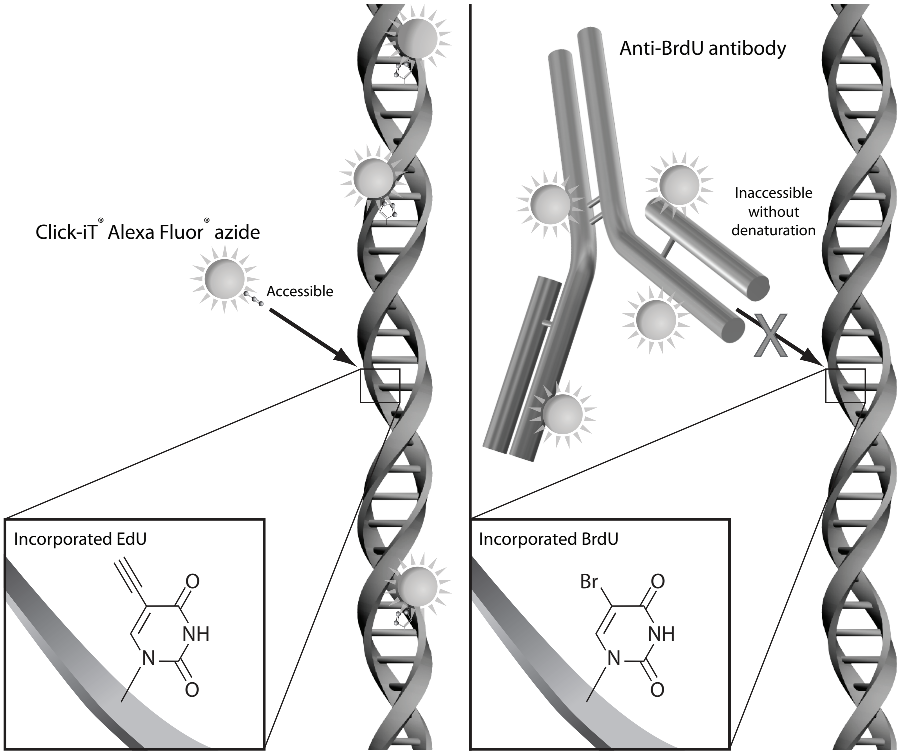

Figure 1. Detection of the incorporated EdU with the Alexa Fluor® azide versus incorporated BrdU with an anti-BrdU antibody. The small size of the Alexa Fluor® azide eliminates the need to denature the DNA for the EdU detection reagent to gain access to the nucleotide.

Table 2. Click-iT® detection reagent compatibility.

Molecule	Compatibility*
Qdot® nanocrystals	Use Qdot® nanocrystals **after** the Click-iT® detection reaction.
Fluorescent proteins such as Green Fluorescent Protein (GFP)	Use organic dye-based reagents, such as TC-FlAsH™ or TC-ReAsH™ reagents, for protein expression detection or anti-GFP rabbit or chicken antibodies **before** the Click-iT® detection reaction.
Organic dyes such as Alexa Fluor® dyes, fluorescein (FITC)	**Completely compatible** with the Click-iT® detection reaction.
TC-FlAsH™ or TC-ReAsH™ reagents	Detect the tetracysteine (TC) tag with FlAsH™ or ReAsH™ reagents **before** the Click-iT® detection reaction.
Phalloidin	Phalloidin staining is **not compatible** with the Click-iT® detection reaction. Use antibodies against other proteins, such as anti-α-tubulin, for visualization of the cytoskeleton.
Horseradish peroxidase (HRP)	Use HRP **after** the Click-iT® detection reaction.
R-phycoerythrin (R-PE) and R-PE-tandems such as Alexa Fluor® 680-R-PE	Use R-PE and R-PE tandems **after** the Click-iT® detection reaction.
Allophycocyanin (APC) and APC-tandems	**Completely compatible** with the Click-iT® detection reaction.

*Compatibility indicates whether the fluorescent molecule itself or the detection method involves components that are unstable in the presence of copper catalyst used for the Click-iT® detection reaction.

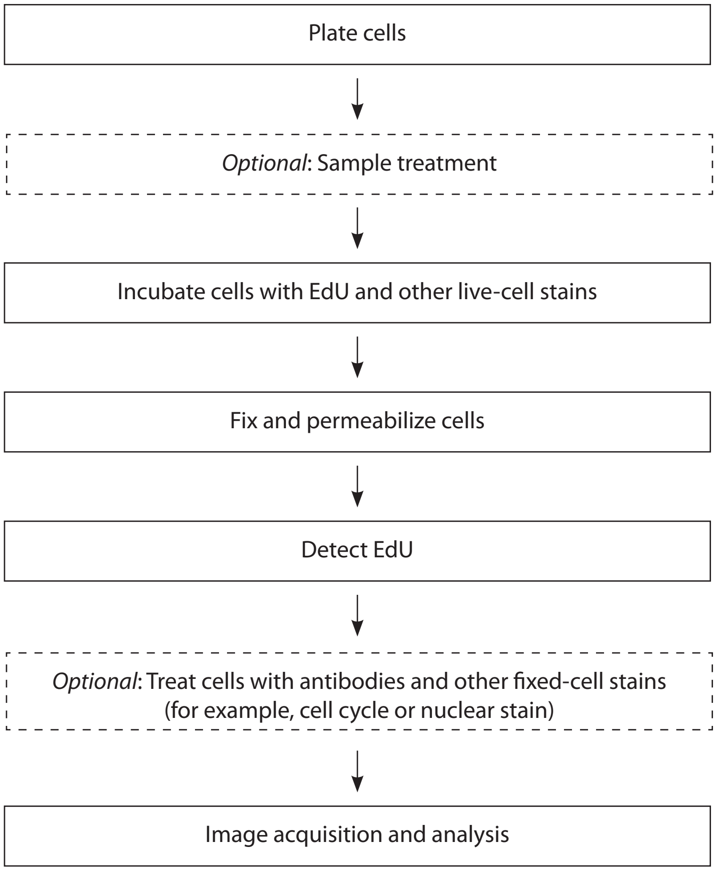

Figure 2. Workflow diagram for the Click-iT® EdU Imaging Assay.

Before You Begin

Materials required but not provided

- Phosphate-buffered saline (PBS, pH 7.2–7.6)
- Fixative (for example, 3.7% Formaldehyde in PBS)
- Permeabilization reagent (for example, 0.5% Triton® X-100 in PBS)
- 3% Bovine serum albumin (BSA) in PBS (3% BSA in PBS), pH 7.4
- Deionized water
- 18 × 18-mm coverslips
- *Optional*: 6-well microplate

Cautions

Hoechst 33342 (Component G) is a known mutagen. Use the dye with appropriate precautions.

DMSO (Component C), provided as a solvent in this kit, is known to facilitate the entry of organic molecules into tissues. Handle reagents containing DMSO using equipment and practices appropriate for the hazards posed by such materials. Dispose of the reagents in compliance with all pertaining local regulations.

Preparing the stock solutions

1.1 Allow the vials to warm to room temperature before opening.

1.2 Prepare a 10-mM stock solution of EdU (Component A): Add 2 mL of DMSO (Component C) or an aqueous solution (for example, buffer, saline) to EdU (Component A), then mix well.

After use, store any remaining stock solution at ≤–20°C. When stored as directed, this stock solution is stable for up to 1 year.

1.3 Prepare a working solution of the Alexa Fluor® azide (Component B): Add 70 µL of DMSO (Component C) to Component B, then mix well.

After use, store any remaining working solution at ≤–20°C. When stored as directed, this working solution is stable for up to 1 year.

1.4 Prepare a working solution of 1X Click-iT® EdU reaction buffer (Component D): Transfer all of the solution (4 mL) in the Component D bottle to 36 mL of deionized water. Rinse the Component D bottle with some of the diluted Click-iT™ EdU reaction buffer to ensure the transfer of all of the 10X concentrate.

To make smaller amounts of 1X Click-iT® EdU reaction buffer, dilute volumes from the Component D bottle 1:10 with deionized water. After use, store any remaining 1X solution at 2–6˚C. When stored as directed, this 1X solution is stable for up to 6 months.

1.5 To make a 10X stock solution of the Click-iT® EdU buffer additive (Component F): Add 2 mL of deionized water to the vial, then mix until fully dissolved. After use, store any remaining stock solution at ≤–20°C.

When stored as directed, this stock solution is stable for up to 1 year. If the solution develops a brown color, it has degraded and should be discarded.

Experimental Protocols

Labeling cells with EdU

The following protocol was developed with A549, HeLa, and NIH/3T3 cells with an optimized EdU concentration of 10 μM, but it can be adapted for any adherent cell type. Growth medium, cell density, cell type variations, and other factors may influence labeling. In initial experiments, we recommend testing a range of EdU concentrations to determine the optimal concentration for your cell type and experimental conditions. Although sufficient material is included with the kit for standard dose response, additional EdU (Cat. nos. A10044, E10187) is available. If you are currently using a BrdU-based assay for cell proliferation, a similar concentration to BrdU is a good starting concentration for EdU.

2.1 Plate the cells on coverslips at the desired density, then allow them to recover overnight before additional treatment.

2.2 Prepare a 2X working solution of EdU (Component A) in complete medium from the 10-mM stock solution. A suggested starting concentration is 10 μM.

2.3 Prewarm the 2X EdU solution, then add an equal volume of the 2X EdU solution to the volume of media containing cells to be treated to obtain a 1X EdU solution. (For example, for a final concentration of 10 μM, replace half of the media with fresh media containing 20 μM of EdU). We do not recommend replacing all of the media, because this could affect the rate of cell proliferation.

2.4 Incubate the cells for the desired length of time under conditions optimal for the cell type. The time of EdU exposure to the cells allows for direct measurement of cells synthesizing DNA. The choice of time points and the length of time depends on the cell growth rate. Pulse labeling of cells by brief exposures to EdU permits studies of cell-cycle kinetics.

2.5 Incubate under conditions optimal for the cell type for the desired length of time. The time of EdU exposure to the cells allows for the direct measurement of cells synthesizing DNA. The choice of time points and length of time for pulsing depends on the cell growth rate.

2.6 Proceed immediately to **Cell fixation and permeabilization** (steps 3.1–3.3), followed by **EdU detection** (steps 4.1–4.7).

Cell fixation and permeabilization

Note: This protocol is optimized with a fixation step using 3.7% formaldehyde in PBS, followed by a 0.5% Triton® X-100 permeabilization step. However, this protocol is also amenable to other fixation/permeabilization reagents, such as methanol and saponin.

Transfer the coverslips into a 6-well plate for convenient processing, such that each well contains a single coverslip.

3.1 After incubation, remove the media and add 1 mL of 3.7% formaldehyde in PBS to each well containing the coverslips. Incubate for 15 minutes at room temperature.

3.2 Remove the fixative and wash the cells in each well twice with 1 mL of 3% BSA in PBS.

3.3 Remove the wash solution. Add 1 mL of 0.5% Triton® X-100 in PBS to each well, then incubate at room temperature for 20 minutes.

EdU detection

Note: This protocol uses 500 µL of Click-iT® reaction cocktail per coverslip. A smaller volume can be used as long as the remaining reaction components are maintained at the same ratios.

4.1 Prepare 1X Click-iT® EdU buffer additive (see Table 3) by diluting the 10X solution (prepared in step 1.5) 1:10 in deionized water. Prepare this solution **fresh** and use the solution on the same day.

4.2 Prepare Click-iT® reaction cocktail according to Table 3. It is important to add the ingredients in the order listed in the table; otherwise, the reaction will not proceed optimally. Use the Click-iT® reaction cocktail within 15 minutes of preparation.

Table 3. Click-iT® reaction cocktails.

Reaction components*	Number of coverslips						
	1	2	4	5	10	25	50
1X Click-iT® reaction buffer (prepared in step 1.4)	430 µL	860 µL	1.8 mL	2.2 mL	4.3 mL	10.7 mL	21.4 mL
$CuSO_4$ (Component E)	20 µL	40 µL	80 µL	100 µL	200 µL	500 µL	1 mL
Alexa Fluor® azide (prepared in step 1.3)	1.2 µL	2.5 µL	5 µL	6 µL	12.5 µL	31 µL	62 µL
Reaction buffer additive (prepared in step 4.1)	50 µL	100 µL	200 µL	250 µL	500 µL	1.25 mL	2.5 mL
Total volume	500 µL	1 mL	2 mL	2.5 mL	5 mL	12.5 mL	25 mL

*__Note__: Add the ingredients in the order listed in the table.

4.3 Remove the permeabilization buffer (step 3.3), then wash the cells in each well twice with 1 mL of 3% BSA in PBS. Remove the wash solution.

4.4 Add 0.5 mL of Click-iT® reaction cocktail to each well containing a coverslip. Rock the plate briefly to insure that the reaction cocktail is distributed evenly over the coverslip.

4.5 Incubate the plate for 30 minutes at room temperature, **protected from light**.

4.6 Remove the reaction cocktail, then wash each well once with 1 mL of 3% BSA in PBS. Remove the wash solution.

For nuclear staining, proceed to **DNA staining**. If no additional staining is desired, proceed to **Imaging and analysis**.

4.7 *Optional*: Perform antibody labeling of the samples at this time, following the recommendations from the manufacturer of the primary and secondary antibody. It is important to keep the samples **protected from light** during incubations.

DNA staining

5.1 Wash each well with 1 mL of PBS. Remove the wash solution.

5.2 Dilute the Hoechst 33342 (Component G) solution 1:2000 in PBS to obtain a 1X Hoechst 33342 solution (the final concentration is 5 µg/mL).

Note: A range between 2–10 µg/mL of Hoechst 33342 has been shown to work.

5.3 Add 1 mL of 1X Hoechst 33342 solution per well. Incubate for 30 minutes at room temperature, **protected from light**. Remove the Hoechst 33342 solution.

5.4 Wash each well twice with 1 mL of PBS. Remove the wash solution.

Imaging and analysis

Click-iT® EdU cells are compatible with all methods of slide preparation, including wet mount or prepared mounting media. See Table 4 for the approximate fluorescence excitation/emission maxima for Alexa Fluor® dyes and Hoechst 33342 dye bound to DNA.

Table 4. Approximate fluorescence excitation/emission maxima.

Fluorophore	Excitation (nm)	Emission (nm)
Alexa Fluor® 488	495	519
Alexa Fluor® 555	555	565
Alexa Fluor® 594	590	615
Alexa Fluor® 647	650	670
Hoechst 33342, bound to DNA	350	461

References

1. Proc Natl Acad Sci USA 105, 2415 (2008); **2.** ChemBioChem 4, 1147 (2003); **3.** J Am Chem Soc 125, 3192 (2003); **4.** Angew Chem Int Ed Engl 41, 2596 (2002); **5.** Angew Chem Int Ed Engl 40, 2004 (2001).

Product List **Current prices may be obtained from our website or from our Customer Service Department.**

	Cat No.	Product Name	Unit Size
Buy Now	C10337	Click-iT® EdU Alexa Fluor® 488 Imaging Kit *for 50 coverslips*	1 kit
Buy Now	C10338	Click-iT® EdU Alexa Fluor® 555 Imaging Kit *for 50 coverslips*	1 kit
Buy Now	C10339	Click-iT® EdU Alexa Fluor® 594 Imaging Kit *for 50 coverslips*	1 kit
Buy Now	C10340	Click-iT® EdU Alexa Fluor® 647 Imaging Kit *for 50 coverslips*	1 kit
	Related Products		
Buy Now	C10289	Click-iT® AHA Alexa Fluor® 488 Protein Synthesis HCS Assay	1 kit
Buy Now	C10327	Click-iT® RNA Alexa Fluor® 488 HCS Assay *2-plate size*	1 kit
Buy Now	C10328	Click-iT® RNA Alexa Fluor® 594 HCS Assay *2-plate size*	1 kit
Buy Now	C10329	Click-iT® RNA Alexa Fluor® 488 Imaging Kit *for 25 coverslips*	1 kit
Buy Now	C10330	Click-iT® RNA Alexa Fluor® 594 Imaging Kit *for 25 coverslips*	1 kit
Buy Now	C10350	Click-iT® EdU Alexa Fluor® 488 HCS Assay *2-plate size*	1 kit
Buy Now	C10351	Click-iT® EdU Alexa Fluor® 488 HCS Assay *10-plate size*	1 kit
Buy Now	C10352	Click-iT® EdU Alexa Fluor® 555 HCS Assay *2-plate size*	1 kit
Buy Now	C10353	Click-iT® EdU Alexa Fluor® 555 HCS Assay *10-plate size*	1 kit
Buy Now	C10354	Click-iT® EdU Alexa Fluor® 594 HCS Assay *2-plate size*	1 kit
Buy Now	C10355	Click-iT® EdU Alexa Fluor® 594 HCS Assay *10-plate size*	1 kit
Buy Now	C10356	Click-iT® EdU Alexa Fluor® 647 HCS Assay *2-plate size*	1 kit
Buy Now	C10357	Click-iT® EdU Alexa Fluor® 647 HCS Assay *10-plate size*	1 kit
Buy Now	A10044	EdU (5-ethynyl-2´-deoxyuridine)	50 mg
Buy Now	E10187	EdU (5-ethynyl-2´-deoxyuridine)	500 mg
Buy Now	E10415	EdU (5-ethynyl-2´-deoxyuridine)	5 g
Buy Now	H3570	Hoechst 33342, trihydrochloride, trihydrate *10 mg/mL solution in water*	10 mL

Contact Information

Corporate Headquarters
5791 Van Allen Way
Carlsbad, CA 92008
USA
Phone: +1 760 603 7200
Fax: +1 760 602 6500
Email: techsupport@lifetech.com

European Headquarters
Inchinnan Business Park
3 Fountain Drive
Paisley PA4 9RF
UK
Phone: +44 141 814 6100
Toll-Free Phone: 0800 269 210
Toll-Free Tech: 0800 838 380
Fax: +44 141 814 6260
Tech Fax: +44 141 814 6117
Email: euroinfo@invitrogen.com
Email Tech: eurotech@invitrogen.com

Japanese Headquarters
LOOP-X Bldg. 6F
3-9-15, Kaigan
Minato-ku, Tokyo 108-0022
Japan
Phone: +81 3 5730 6509
Fax: +81 3 5730 6519
Email: jpinfo@invitrogen.com

Additional international offices are listed at **www.lifetechnologies.com.**

These high-quality reagents and materials must be used by, or directly under the supervision of, a technically qualified individual experienced in handling potentially hazardous chemicals. Read the Safety Data Sheet provided for each product; other regulatory considerations may apply.

Obtaining Support
For the latest services and support information for all locations, go to **www.lifetechnologies.com**.

At the website, you can:

- Access worldwide telephone and fax numbers to contact Technical Support and Sales facilities
- Search through frequently asked questions (FAQs)
- Submit a question directly to Technical Support (techsupport@lifetech.com)
- Search for user documents, SDSs, vector maps and sequences, application notes, formulations, handbooks, certificates of analysis, citations, and other product support documents
- Obtain information about customer training
- Download software updates and patches

SDS
Safety Data Sheets (SDSs) are available at **www.lifetechnologies.com/sds**.

Certificate of Analysis
The Certificate of Analysis provides detailed quality control and product qualification information for each product. Certificates of Analysis are available on our website. Go to **www.lifetechnologies.com/support** and search for the Certificate of Analysis by product lot number, which is printed on the product packaging (tube, pouch, or box).

Limited Warranty
Invitrogen (a part of Life Technologies Corporation) is committed to providing our customers with high-quality goods and services. Our goal is to ensure that every customer is 100% satisfied with our products and our service. If you should have any questions or concerns about an Invitrogen product or service, contact our Technical Support Representatives.
All Invitrogen products are warranted to perform according to specifications stated on the certificate of analysis. The Company will replace, free of charge, any product that does not meet those specifications. This warranty limits the Company's liability to only the price of the product. No warranty is granted for products beyond their listed expiration date. No warranty is applicable unless all product components are stored in accordance with instructions. The Company reserves the right to select the method(s) used to analyze a product unless the Company agrees to a specified method in writing prior to acceptance of the order.
Invitrogen makes every effort to ensure the accuracy of its publications, but realizes that the occasional typographical or other error is inevitable. Therefore the Company makes no warranty of any kind regarding the contents of any publications or documentation. If you discover an error in any of our publications, please report it to our Technical Support Representatives.
Life Technologies Corporation shall have no responsibility or liability for any special, incidental, indirect or consequential loss or damage whatsoever. The above limited warranty is sole and exclusive. No other warranty is made, whether expressed or implied, including any warranty of merchantability or fitness for a particular purpose.

Limited Use Label License: Research Use Only
The purchase of this product conveys to the purchaser the limited, non-transferable right to use the purchased amount of the product only to perform internal research for the sole benefit of the purchaser. No right to resell this product or any of its components is conveyed expressly, by implication, or by estoppel. This product is for internal research purposes only and is not for use in commercial services of any kind, including, without limitation, reporting the results of purchaser's activities for a fee or other form of consideration. For information on obtaining additional rights, please contact outlicensing@lifetech.com or Out Licensing, Life Technologies Corporation, 5791 Van Allen Way, Carlsbad, California 92008.

The trademarks mentioned herein are the property of Life Technologies Corporation or their respective owners.

©2011 Life Technologies Corporation. All rights reserved.

For research use only. Not intended for any animal or human therapeutic or diagnostic use.
